# COVID-19’s Impact on Farmers Market Sales in the Washington, D.C., Area

**DOI:** 10.1017/aae.2020.37

**Published:** 2021-01-13

**Authors:** Jeffrey K. O’Hara, Timothy A. Woods, Nony Dutton, Nick Stavely

**Affiliations:** 1 Agricultural Marketing Service, U.S. Department of Agriculture, Washington, DC, USA; 2 Department of Agricultural Economics, University of Kentucky, Lexington, KY, USA; 3 FRESHFARM, Washington, DC, USA

**Keywords:** COVID-19, data collection, farmers markets, local foods, Q10, Q12, Q13

## Abstract

We use a sales database of farmers market vendors in the Washington, D.C., area to estimate how first half 2020 sales were impacted by the coronavirus (COVID-19) outbreak. We use 2019 data as a counterfactual for sales that would have occurred in 2020 in the absence of COVID-19. For neighborhood weekend markets that were able to remain open during the pandemic, the change in 2020 average sales between the winter and spring is between 75% and 79% lower than in 2019. Other farmers markets, particularly weekday markets in business districts, experienced delayed openings or were closed for the entire year.

## 1. Introduction

The U.S. Department of Agriculture’s (USDA) Census of Agriculture data indicates that direct-to-consumer (DTC) food sales by the U.S. farms doubled between 1992 and 2012, and most recently equaled $3 billion in 2017 (O’Hara and Benson, 2019).^1^ DTC outlets include on-farm stores, farmers markets, roadside stands, community-supported agriculture (CSA) programs, and online marketplaces. Still, the size of DTC market activity is larger than USDA’s Census of Agriculture estimates. This is because nonfarm food businesses, along with farms and ranches, have been increasingly using farmers markets to develop new product offerings, establish marketing strategies, and perhaps increase in size (Low et al., 2020; McDonald, 2019; O’Hara, 2020; O’Hara, Castillo, and McFadden, 2020). Further, the localized economic impacts from farmers market sales can exceed those from traditional food retailers (Hughes et al., 2008; Hughes and Isengildina-Massa, 2015).

However, a lack of real-time market data implied that throughout 2020, public narratives about how the coronavirus (COVID-19) was impacting the U.S. local food sector were speculative, anecdotal, and dissonant with each other (Lusk, 2020). On the one hand, some claimed that local food systems are more resilient than conventional supply chains to COVID-19 disruptions because they are decentralized and have fewer intermediaries (Colicchio and Kessler, 2020; Pollan, 2020; Thilmany et al., 2020a). On the other hand, others projected devastating financial hardship to local producers if direct sales via farmers markets, restaurants, and institutions collapsed and the economy went into a major recession (resourcED, 2020; Thilmany et al., 2020b). These contradictory assessments presented a challenge to policy makers helping the agricultural sector adapt to COVID-19, since officials were creating large emergency support programs within a brief time frame (Johansson et al., 2020).

We provide one of the first estimates of how COVID-19 impacted U.S. local food sales. To do so, we use a sales database maintained by FRESHFARM, which is an organization that manages farmers markets in the Washington, D.C., area. FRESHFARM’s database records sales from every vendor at each FRESHFARM farmers market for each market day. The central hypothesis that we test, using differences-in-differences (DD) regressions, is how average vendor-level sales changed in 2020 between the March 8–July 4 and January 1–March 7 time intervals, relative to the corresponding change in 2019 sales among the same set of market-weeks.^2^ We refer to the former time period as the “spring” and latter time period as the “winter” for accessibility throughout the paper, even though these time periods do not correspond exactly to these seasons. Our research represents the first analysis of FRESHFARM’s sales data, and we exemplify insights that could be attained elsewhere if data collection by farmers market organizations expanded.

Few studies have explored how COVID-19 impacted the local foods sector using practitioner-collected data. Thilmany et al. (2020a) undertook semi-structured interviews of e-commerce operators in one of the first economic studies that examined how COVID-19 impacted the U.S. local food sector. Pre-pandemic studies of farmers market vendors have predominately used cross-sectional survey data collected by either USDA (e.g., O’Hara and Lin, 2020; O’Hara and Low, 2020) or researchers (e.g., Henneberry, Whitacre, and Agustini, 2009; Hughes et al., 2008; Hughes and Isengildina-Massa, 2015; Varner and Otto, 2008). Archambault et al. (2020) is one of the few studies to analyze sales data collected by a farmers market organization.

**Figure 1.**
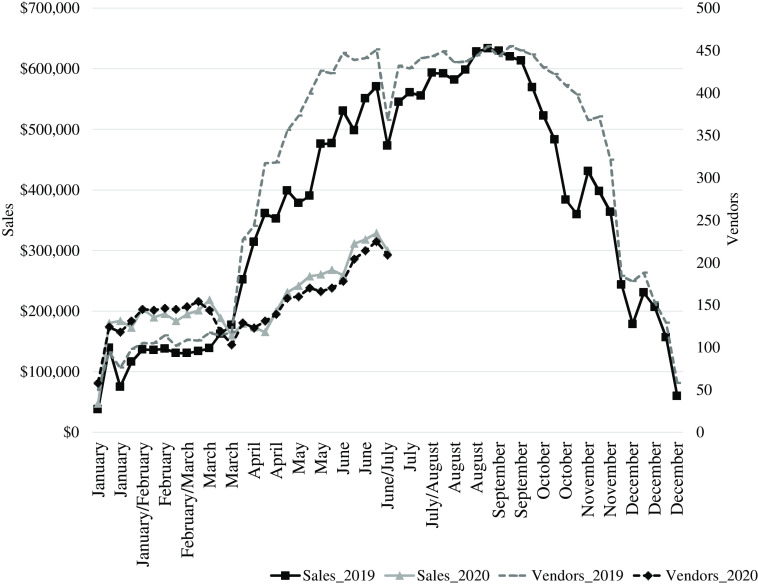
Year-over-year weekly sales and vendors at all FRESHFARM markets.

Our study contributes to the COVID-19 local foods literature by studying farmers markets, which are a prominent DTC market channel. In contrast, Thilmany et al. (2020a) focused on online sales, which were modest for DTC vendors prior to 2020 (O’Hara and Low, 2020). Also, unlike studies of farmers market vendors that have used cross-sectional data, our analysis of FRESHFARM’s intra-annual data represents one of the few studies that have examined year-over-year trends in farmers market sales. Using 2019 sales data as a counterfactual scenario is important because COVID-19 did not impact daily life in the U.S. in 2020 until mid-March, and intra-annual fluctuations in sales occur regardless due to changes in the growing season (Figure 1). Further, whereas Archambault et al. (2020) modeled aggregate weekly sales at one farmers market, we examine sales at the vendor level across FRESHFARM’s network of diverse farmers markets. So, we (a) examine sales fluctuations across vendors depending on the types of products that they sell and (b) distinguish between the different impacts that COVID-19 had on weekend markets in neighborhoods and weekday markets in business districts.

## 2. Background

### 2.1. Farmers Markets and COVID-19

The COVID-19 pandemic impacted both the supply and demand for food from DTC vendors. In March and April 2020, the demand for food simultaneously surged at supermarkets and deteriorated at restaurants (Lusk and McCluskey, 2020). The increase in demand for at-home foods could conceivably benefit DTC producers, since consumers became increasingly interested in finding local foods in March 2020 as shortages occurred at supermarkets (Schmidt et al., 2020).

However, some farmers markets were shuttered altogether; in Vermont, for instance, they were closed statewide from March to May. Other farmers markets had to adopt exacting social distancing guidelines in order to remain open during the pandemic. While these protocols evolved throughout 2020 and the details varied by jurisdiction, they included requiring customers to pre-order food online for on-site pickup, requiring vendors to sell prepackaged foods, requiring attendees to wear masks and maintain distances from others inside the market, eliminating on-site food preparation and sampling, canceling community events, restricting the number of shoppers inside the market at any given time by requiring a single point-of-entry, and encouraging shoppers to reserve timeslots for pickup to balance attendance throughout the day (Klisch and Soule, 2020).

Maryland, Virginia, and Washington, D.C., each provided a pathway for FRESHFARM to keep its farmers markets open with social distancing procedures (online supplementary Appendix 1). However, these protocols were demanding to implement. At the Dupont Circle farmers market, for instance, FRESHFARM needed 10 or 11 market managers each Sunday during the spring of 2020 to implement a single point-of-entry system. Because only one entry point existed, shoppers waited 45 minutes on average to enter the Dupont Circle market and the line outside the market extended for five blocks. Further, we describe subsequently that other pandemic-related reasons prevented FRESHFARM from keeping some of its markets open.

These social distancing regulations imposed high transaction costs on DTC producers, in addition to market managers. DTC producers needed an online marketplace to adhere to the abruptly established pre-ordering requirements, and only 8% of DTC farms had an online marketplace as of 2015 (O’Hara and Low, 2020). Some DTC producers could not attend farmers markets altogether even when the markets remained open. This was attributable to market managers reducing the number of vendors for social distancing purposes, vendor’s health challenges, and labor shortfalls (FMC, 2020). Local vendors also couldn’t necessarily increase supply quickly due to capacity constraints. For instance, the closure of meatpacking plants across the U.S. due to COVID-19 outbreaks led to reductions in supply at traditional retail stores (Tonsor and Schulz, 2020), and consumers sought alternate outlets. However, niche meat processors experienced backlogs in processing livestock from local farmers (Richards and Vassalos, 2020).

### 2.2. FRESHFARM’s Database

FRESHFARM’s sales database is unique among the U.S. farmers market associations due to its size, as well as the frequency and granularity at which they collect sales data (O’Hara and Stavely, 2020). Only 11% of the 8,140 U.S. farmers markets in 2019 collected sales information from vendors (USDA NASS, 2020). It is challenging for farmers markets to collect sales data from vendors due to capacity constraints, staff turnover among volunteer market managers, and the reluctance vendors may have in providing sales data to market staff (Wilson et al., 2018; Wolnik, Cheek, and Weaver, 2019).

Both market managers and vendors enter data into FRESHFARM’s Google Sheets database, *Market Tracker*. FRESHFARM’s managers reconcile the redemption of benefit and incentive vouchers with each vendor onsite at the market, and then enter the data subsequently. Vendors enter daily sales data from cash, credit, and pre-ordered online transactions into the *Market Tracker* on a monthly basis. FRESHFARM does not subsequently verify the vendors’ entries. While vendors’ fees to FRESHFARM are a percentage of their sales, which provides an incentive to underreport sales, FRESHFARM has not identified irregular reporting patterns that suggest fabricated entries. Further, vendors may accurately report sales because: (a) FRESHFARM uses the data to obtain funding that benefits vendors, (b) some vendors use the database for financial record-keeping and/or to evaluate their market performance, and (c) FRESHFARM needs the dues to operate the markets on behalf of vendors.

FRESHFARM’s data has four attributes that survey data does not provide. First, FRESHFARM collects data on an ongoing basis and it is available in real time. In contrast, USDA collects local food data intermittently and releases it with a time lag. Second, FRESHFARM’s sales data is disaggregated at both the vendor level and market level. Alternately, USDA’s surveys either (a) collect sales data from direct marketing farmers that cannot be linked to specific farmers markets (O’Hara and Lin, 2020) or (b) collect aggregated market-level data from farmers market managers that cannot be linked to specific vendors (USDA NASS, 2020). Third, FRESHFARM measures within-year seasonal sales fluctuations with daily data. Intra-annual sales trends are unavailable from USDA annual survey data, as well as from sales data collected in cross-sectional surveys. Fourth, FRESHFARM tracks sales from all types of farmers market vendors, including farmers, fishers, food manufacturers, beverage and alcohol producers, and nonfood producers. USDA collects DTC sales data of edible food products from farms and ranches exclusively, which constituted only 56% of 2019 sales at FRESHFARM’s markets.

FRESHFARM staff replaced the actual vendor names in the database with random identification numbers prior to sharing the data externally. This step is akin to de-identification processes that other institutions implement when allowing researchers to access confidential microdata, including USDA. FRESHFARM does not collect sales data disaggregated by specific products. However, one vendor-specific attribute that FRESHFARM included in the database for this research are indicator variables that correspond to the type of product that each vendor predominately sells. With FRESHFARM’s classification scheme, each vendor is assigned to one (and only one) product type.

### 2.3. FRESHFARM’s Farmers Markets

FRESHFARM is the third largest U.S. farmers market association after GrowNYC and the Pacific Coast Farmers Market Association. FRESHFARM, in 2019, managed 30 farmers markets in the Washington, D.C., area that collectively grossed $19.4 million in sales. There were 269 distinct vendors that sold at FRESHFARM’s markets in 2019 and the first half of 2020.

Seven of FRESHFARM’s markets had opened for the 2020 season prior to the onset of the COVID-19 pandemic: Arlington Courthouse, Columbia Heights—Saturday, Dupont Circle, Monroe Street, Mosaic, Oakton, and Silver Spring (Table 1). These seven markets each (a) occur on weekends, (b) are predominately attended by neighborhood residents that walk to the market to purchase food for at-home consumption, (c) are relatively large, as they collectively accounted for 75% of FRESHFARM’s first half sales in 2019, and (d) are located in higher income neighborhoods, which we document in Table 1.

**Table 1.** FRESHFARM’s farmers markets

Market	Day of week	Location	Nominal 2018 Med. HH Inc. (Zip Code)	2019 opening date	2020 opening date
Arlington Courthouse	Saturday	Arlington, VA	$125,363	January 5	January 4
Ballston	Thursday	Arlington, VA	$103,654	April 4	June 18
Capital Riverfront	Sunday	Washington, D.C.	$126,323	May 5	Canceled
Cesar Chavez	Saturday	Washington, D.C.	$36,398	June 1	July 18
Chantilly	Thursday	Chantilly, VA	$124,258	May 9	Canceled
CityCenterDC	Tuesday	Washington, D.C.	$109,318	June 5	Canceled
Columbia Heights	Saturday	Washington, D.C.	$88,463	February 2	February 1
Columbia Heights	Wednesday	Washington, D.C.	$88,463	May 22	July 15
Crystal City	Tuesday	Arlington, VA	$110,817	May 14	June 16
Dupont Circle	Sunday	Washington, D.C.	$88,553	January 6	January 5
Foggy Bottom	Wednesday	Washington, D.C.	N.A.	April 3	Canceled
Fort Totten	Tuesday	Washington, D.C.	$71,569	June 4	Canceled
Gainesville	Sunday	Gainesville, VA	$131,184	April 28	Canceled
Glover Park—Burleith	Saturday	Washington, D.C.	$130,920	May 18	Canceled
H Street NE	Saturday	Washington, D.C.	$84,892	April 6	April 4
Kenilworth Rec. Center	Saturday	Washington, D.C.	$36,398	June 1	July 18
Minnesota Avenue	Thursday	Washington, D.C.	$36,398	June 6	July 16
Monroe Street	Saturday	Washington, D.C.	$77,560	April 20	March 7
Mosaic	Sunday	Fairfax, VA	$108,632	April 6	January 5
Mount Vernon Triangle	Saturday	Washington, D.C.	$109,318	May 4	May 16
NoMa	Sunday	Washington, D.C.	$84,892	May 5	Canceled
Oakton	Saturday	Oakton, VA	$161,333	January 5	January 4
Penn Quarter	Thursday	Washington, D.C.	$147,522	April 4	June 11
Reston	Wednesday	Reston, VA	$116,842	April 17	June 17
Rosslyn	Wednesday	Arlington, VA	$115,292	May 22	June 3
Silver Spring	Saturday	Silver Spring, MD	$84,645	January 5	January 4
Springfield	Saturday	Springfield, VA	$99,566	April 20	May 2
The Boro	Saturday	McClean, VA	$115,099	Not Open	July 30
Tysons	Sunday	McClean, VA	$115,099	June 2	Canceled
Uptown	Saturday	Washington, D.C.	$71,569	April 20	April 18
White House	Thursday	Washington, D.C.	N.A.	April 4	Canceled

MD, Maryland; VA, Virginia.

Notes: The U.S. median household income in 2018 was $61,937 (Census Bureau, 2020).

Orchard and produce farms accounted for 42% of first half 2019 sales at these seven markets (Table 2). Orchard farms in the Washington, D.C., area predominately sell apples, pears, peaches, and associated value-added products like cider. Produce farms predominately sell berries and vegetables. Nonfarm vendors are also prominent at these seven markets. Baked good vendors had the second highest level of sales, by product type, in the winter periods. The prominence of baked good vendors at FRESHFARM’s markets is consistent with national-level trends on the increasing role they have at farmers markets (O’Hara, Castillo, and McFadden, 2020). Sales by hot prepared food vendors (e.g., ready-to-eat sandwiches, pizza, or tacos) and non-pickle value-added food vendors (e.g., hot sauce, jam, jelly, or dried fruit slices) were each almost equal to meat vendor sales during the spring of 2019.

**Table 2.** Year-over-year sales comparisons by vendor and farmers market type

Vendor Type	Seven neighborhood markets						Other markets		
	Winter			Spring			Spring		
	2019	2020	% Change	2019	2020	% Change	2019	2020	% Change
Orchard	$275	$367	33%	$1,051	$899	−14%	$173	$64	−63%
Produce	$193	$282	46%	$932	$736	−21%	$345	$147	−57%
Meat	$142	$199	41%	$386	$541	40%	$132	$27	−79%
Baked Goods	$203	$309	53%	$654	$436	−33%	$235	$25	−89%
Value-Added Foods	$94	$144	54%	$371	$223	−40%	$194	$48	−75%
Dairy Products	$61	$88	45%	$263	$285	8%	$49	$14	−72%
Prepared (Hot) Foods	$73	$187	155%	$363	$138	−62%	$529	$30	−94%
Plants	$0	$1	N.A.	$152	$97	−36%	$5	$0	−100%
Alcohol	$31	$38	22%	$85	$93	9%	$44	$2	−96%
Pickles and Ferments	$53	$73	38%	$193	$92	−53%	$74	$6	−92%
Seafood	$17	$21	24%	$49	$83	68%	$10	$6	−35%
Other	$31	$42	37%	$117	$57	−51%	$91	$9	−90%
Total	$1,172	$1,751	49%	$4,618	$3,680	−20%	$1,880	$379	−80%

Notes: Sales reported in $000. “Other” products include coffee, dry goods, value-added beverages (e.g., smoothies), and nonfoods.

FRESHFARM’s other 23 markets had not yet opened for the 2020 season prior to the pandemic’s onset. Twelve of these seasonal markets occur on weekdays, and the other 11 markets operate on weekends in residential neighborhoods. FRESHFARM’s weekday markets predominately occur in high foot traffic locations that are convenient for professional-class commuters. While FRESHFARM’s 3 largest markets occur on the weekend, 5 of these business district markets were among FRESHFARM’s 10 highest-grossing markets in the first half of 2019: Ballston, CityCenterDC, Foggy Bottom, Penn Quarter, and White House.

Vendors that sold hot prepared foods made 28% of the first half 2019 sales at these seasonal markets (Table 2). Unlike FRESHFARM’s large weekend markets, orchard farms had the fifth highest level of first half sales at FRESHFARM’s other markets in 2019, since they were exceeded by sales from prepared food, baked good, produce, and value-added food vendors. Sales from meat and dairy vendors only accounted for 10% of 2019 sales at FRESHFARM’s other markets.

## 3. Methods

### 3.1. Conceptual Overview

We estimate how the COVID-19 pandemic impacted FRESHFARM’s vendors by comparing sales between 2019 and 2020. Typically in DD models, researchers compare observations measured at the same points in time across different areas. A more traditional DD model is infeasible for our purposes since: (a) there aren’t many, if any, U.S. farmers markets that were unaffected by COVID-19, (b) comparable data from markets in other U.S. cities is not readily available, and (c) seasonal fluctuations in farmers markets sales elsewhere in the U.S. are not necessarily the same as in Washington, D.C., due to differences in growing seasons. For orientation, the states (Pennsylvania and New Jersey) and time intervals in Card and Krueger (1994) are equivalent, respectively, to years (2019 and 2020) and market-weeks in our regressions.

Our “common trends” identification assumption is that FRESHFARM’s 2019 sales trends are a realistic counterfactual for 2020 sales trends that would have occurred in the absence of COVID-19. The merits of using 2019 sales data as a control group, instead of older years, are that the composition of farmers market vendors and the socioeconomic profiles of shoppers in 2019 more closely resemble 2020 baseline conditions than older years. Also, FRESHFARM’s sales in 2019 increased considerably over 2018 sales levels, which could be indicative of an increasing demand for products at FRESHFARM’s markets by neighborhood residents.

We focus on the seven neighborhood markets open prior to the pandemic in the DD model. These seven markets accounted for 93% of FRESHFARM’s first half sales in 2020. We focus on these markets because (a) these are the only markets for which we can measure “pre-treatment” winter sales in 2020 and (b) FRESHFARM was able to keep these markets open in 2020 by adopting strict social distancing protocols. In only one instance—the Courthouse market on March 21—did any of these markets close for a market-week during 2020 because of COVID-19. In contrast, many of FRESHFARM’s other markets either experienced delayed openings or were canceled for the entire year (Table 1). So, the negative impact of COVID-19 on FRESHFARM’s 23 other markets is self-evident, as their first half year-over-year sales declined by 80% (Table 2). Also, the impacts of COVID-19 on these seven neighborhood weekend markets are more likely to be representative of COVID-19’s impacts on farmers markets elsewhere. This is because 63% of the U.S. farmers markets operate on the weekend (USDA NASS, 2020), and it is less feasible to maintain farmers markets oriented to commuters in settings without as much foot traffic as D.C.’s business district areas.

### 3.2. Model

The advantages of estimating DD impacts with regressions are that researchers can (a) use data from multiple vendors and time periods and (b) perform statistical inference on the parameter estimates (Angrist and Pischke, 2009, 2015). We estimate DD regressions using ordinary least squares of the form represented in equation (1):(1)
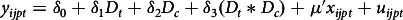



The dependent variable (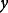) represents the natural log of farmers market sales plus $1 for each vendor (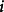), farmers market (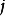), time period (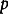), and year (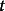). The independent variable 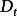 equals one for observations that occur in 2020 and zero for 2019 observations, and the coefficient 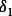 is the difference in average winter sales between 2020 and 2019. The dummy variable 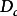 equals one for both 2019 and 2020 observations for the market-weeks corresponding to the spring period that we have defined previously. The coefficient 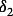
is the difference in 2019 average sales between the spring and winter periods. The interaction of these two terms is an indicator variable that is equal to one for the COVID-19 pandemic weeks in 2020 only, and equal to zero otherwise. The coefficient 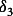 equals the difference between the change in average sales between the spring and winter in 2020 and the corresponding change in average 2019 sales. The independent variables in 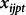 represent control variables. We cluster robust standard errors at the vendor level.

We classify market-weeks that concluded by March 7, 2020 as the “pre-treatment” winter period and market-weeks that began after March 8, 2020 as the “post-treatment” spring period. We classify the week of March 8 in the “post-treatment” spring period since COVID-19 was influencing consumer shopping behavior by that point (Schmidt et al., 2020), D.C.’s first COVID-19 cases were confirmed on March 7, and D.C. office closings began that week. Our results are similar if we instead use the week of March 15 as the first week in the spring period.

We include vendor, farmers market, and product type fixed effects to control for time-invariant unobserved characteristics specific to individual vendors, markets, and product types, respectively. The fixed effects variable for a given vendor is equal to one for sales that occur from that vendor and equal to zero for sales from other vendors. The farmers market and product type fixed effects terms have similar interpretations. We create three grouped product type indicator variables: F&V (orchard and produce), animal (meat, dairy, and seafood), and processed (baked goods, value-added foods, prepared foods, and pickles). Thus, these product type variables terms are interpreted relative to sales from the omitted product types (alcohol, plants, and “other” as defined in Table 2).

### 3.3. Time Periods

In specification I, the time period subscript 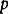 in equation (1) corresponds to two periods: the winter and spring. Thus, specification I is similar to the two-period model in Card and Krueger (1994). Sales for a given period and year, for each vendor at each farmers market, represents the average of sales across the market-weeks within that time period and year. One advantage of a specification with two time periods is that there may be unobserved factors that influence sales on a short-term weekly basis, and some of this weekly volatility might be smoothed out by examining average sales over a longer time period.

In specifications II–V, the time period subscript 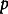 in equation (1) corresponds to market-weeks. An advantage of weekly data is that we control for three factors that are likely to influence weekly sales. Specifically, we include a variable that is equal to the value of the corresponding market-week of the year. This market-week variable is a time trend, and controls for the positive first half sales trend visible in Figure 1. We also control for average temperature and total precipitation levels on the day of the market. We control for these factors since daily weather conditions can influence whether shoppers attend the market and the length of their visit, which in turn influences their expenditures (NOAA NCEI, 2020).

### 3.4. Vendor Attendance at FRESHFARM’s Markets

Properly accounting for vendor nonattendance at markets is important due to the positive correlation between sales and the number of vendors that sell each week and because FRESHFARM experienced a reduction in both sales and vendors in the spring of 2020 relative to the spring of 2019 (Figure 1). In this paper, we use the term “vendor” to refer to food and farm businesses (i.e., not people), so vendors can sell at more than one market per day.

We assign a sales value of $0 to observations in market-weeks when a vendor did not attend an individual market when it was open, and treat sales values as missing for vendors during the weeks that the market was closed. However, we only do this for the markets that an individual vendor attended at least once during the study period. If a vendor never attended a market throughout the study period, then we treat all of their sales values for that market as missing.

There are 72,954 possible opportunities for weekly vendor sales at a farmers market with 193 vendors at 7 farmers markets for 2 years (2019 and 2020) over a time period of 27 weeks. As we mentioned previously, the 27 weeks correspond to the first partial week of January (“market-week zero”) and the subsequent 26 complete weeks. We arrive at 14,692 observations after accounting for the issues that (a) vendors did not sell at each of the markets and (b) not all of the markets were open throughout that entire study period. Of these observations, 7,566 occurred from vendors attending these seven markets when they were open and 7,126 occurred after accounting for vendor nonattendance in the process that we outlined in the previous paragraph. We follow this process for addressing vendor nonattendance because, when estimating DD models, it is important that the composition of the control and treatment groups do not change due to treatment (Angrist and Pischke, 2009).^3^

### 3.5. Sensitivity Tests

In specification III, we include a weekly linear time trend that is interacted with the 2020 indicator variable, 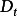. While we assume that sales trends in 2019 and 2020 are parallel in specification II, we relax this assumption in specification III. Controlling for year-specific sales trends is a robustness check against the possibility that the COVID-19 coefficient could be spuriously statistically significant in specification II if we incorrectly assume that trends are parallel when they are not. In specification III, the COVID-19 coefficient will capture deviations in trends that are smooth but need not be parallel (Angrist and Pischke, 2009 & 2015).

In specification IV, we estimate regressions in which we include interactions of the three grouped product terms with the COVID-19 indicator variable. Including these three interaction terms changes the interpretation of four other variables in specification IV: (a) the COVID-19 coefficient represents the sales of the omitted product type (alcohol/plants/other) during COVID-19 and (b) the three grouped product terms (F&V, Animal, Processed) represent non-COVID-19 sales from those respective vendors. Thus, these seven variables (the three interaction terms that we added to specification IV and the four aforementioned variables) are interpreted relative to sales from alcohol/plants/other vendors during the non-COVID-19 time period.

In specification V, we examine whether COVID-19’s impact on sales varied throughout the pandemic. Instead of including the 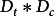 interaction term as in specifications I–IV, we include three interaction terms that represent the impacts COVID-19 had on sales throughout the first half of 2020 at different points in time. The “Year 2020 × Weeks 10–15” variable is the interaction of 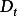
and an indicator variable equal to one for the weeks corresponding to market-weeks 10–15 (i.e., the March 8–April 18 time frame in 2020). The market-week 16–21 and 22–26 terms correspond to the April 19–May 30 and May 31–July 4 time frames, respectively.

### 3.6. Descriptive Statistics

Average sales per market day equals $764, with a maximum of almost $23,000 (Table 3). Sixty-eight percent of the observations occur during the spring period, 52% occur in 2020, and 35% occur during the pandemic. FRESHFARM’s three largest farmers markets (Arlington Courthouse, Dupont Circle, and Silver Spring) are among the seven markets that we examine, and these three markets account for 69% of the observations. The most common product types are produce (19%), value-added foods (13%), and prepared foods (13%).

**Table 3.** Descriptive statistics

Variable	Mean	Std. Dev.	Min.	Max.
Sales ($)	763.74	1,425.87	0	22,885.96
Nat. Log (Sales + $1)	3.47	3.50	0	10.04
Spring Period Indicator	0.68	0.47	0	1
Year 2020 Indicator	0.52	0.50	0	1
COVID-19 Indicator	0.35	0.48	0	1
Precip. (in.)	0.12	0.23	0	1.23
Ave. Temp. (F)	57.57	15.64	26	86
Vendor Type				
Orchard	0.05	0.23	0	1
Produce	0.19	0.39	0	1
Meat	0.08	0.27	0	1
Baked Goods	0.10	0.30	0	1
Value-Added Foods	0.13	0.34	0	1
Dairy Products	0.07	0.25	0	1
Prepared (Hot) Foods	0.13	0.34	0	1
Plants	0.03	0.17	0	1
Alcohol	0.06	0.24	0	1
Pickles and Ferments	0.05	0.21	0	1
Seafood	0.02	0.14	0	1
Other Products	0.09	0.29	0	1
Farmers Market				
Columbia Heights	0.07	0.25	0	1
Dupont Circle	0.28	0.45	0	1
Silver Spring	0.23	0.42	0	1
Mosaic	0.13	0.33	0	1
Oakton	0.07	0.26	0	1
Courthouse	0.18	0.38	0	1
Monroe Street	0.04	0.21	0	1

## 4. Results

### 4.1. Aggregate COVID-19 Impacts

The aggregate sales in Table 2 document the seasonal changes that occurred at FRESHFARM’s markets. At the neighborhood markets, year-over-year winter sales increased from $1.17 million in 2019 to $1.75 million in 2020, which is a difference of $579,000 (49%).^4^ Spring sales declined from $4.62 million in 2019 to $3.68 million in 2020, which is a difference of −$937,000 (20%). The difference between the second difference (−$937,000) and the first difference ($579,000) is −$1.52 million. So, if we disregard the possibility of omitted variable bias, this calculation implies that spring 2020 sales at these seven neighborhood markets were $1.52 million lower than they would have been without COVID-19.

Although not the focus of the empirical model, year-over-year first half sales at FRESHFARM’s other 23 markets declined by $1.5 million in 2020. Thirty-three percent of this decline is attributable to the collapse in sales from prepared food vendors (Table 2). Prepared food vendors were impeded by shelter-in-place orders that encouraged teleworking, and thus led to the shuttering of important business district markets. For instance, the White House market was FRESHFARM’s fourth largest market in 2019, and it was canceled in 2020. Orchard and produce vendors experienced relatively smaller year-over-year declines in sales of 63% and 57%, respectively, at these markets than most other vendor types. This may be because some of these other 23 markets include smaller weekend markets in neighborhoods, and customers made purchases of fruits and vegetables for at-home consumption at them.

### 4.2. Regression Results^5^

In specification I, the adjusted *R* ^2^ of the DD model equals 0.42 (Table 4). The COVID-19 coefficient implies that the change in 2020 average sales between spring and winter periods is 75% lower than the corresponding change in 2019 (*P* < 0.01). The year 2020 coefficient indicates that average 2020 winter sales exceed 2019 winter sales by 79% (*P* < 0.01). The spring coefficient implies that the average spring 2019 sales exceeded winter 2019 sales by 530% (*P* < 0.01).

**Table 4.** Differences-in-differences (DD) regression results

Specification	I	II	III	IV	V
Observations included	Two-period average sales	Weekly sales	Weekly sales	Weekly sales	Weekly sales
Intercept	0.89	0.01	−0.09	−0.02	−0.12
	(0.59)	(0.57)	(0.57)	(0.59)	(0.58)
Year 2020 Indicator	0.58***	0.57***	0.78***	0.56***	0.56***
	(0.22)	(0.17)	(0.20)	(0.18)	(0.17)
Spring Indicator	1.84***	0.30**	−0.01	0.29**	0.13
	(0.23)	(0.15)	(0.12)	(0.15)	(0.12)
COVID-19 (Spring 2020) Indicator	−1.40***	−1.58***	−1.01***	−1.50***	
	(0.26)	(0.22)	(0.20)	(0.40)	
Temp. (F)		0.015***	0.015***	0.015***	0.017***
		(0.003)	(0.003)	(0.003)	(0.003)
Precip. (in.)		−0.23***	−0.28***	−0.23***	−0.26***
		(0.08)	(0.08)	(0.08)	(0.08)
Mkt. Week (time trend)		0.05***	0.07***	0.05***	0.05***
		(0.01)	(0.01)	(0.01)	(0.01)
F&V	2.72***	4.10***	4.10***	3.91***	4.10***
	(0.80)	(0.68)	(0.68)	(0.72)	(0.68)
Animal	2.05***	6.71***	6.71***	6.09***	6.71***
	2.03 E-12	(0.68)	(0.68)	(0.72)	(0.68)
Processed	0.22	−0.50	−0.50	−0.14	−0.50
	(0.80)	(0.68)	(0.68)	(0.66)	(0.68)
Year 2020 × Mkt. Week			−0.04***		
			(0.01)		
F&V × COVID-19				0.27	
				(0.46)	
Animal × COVID-19				1.02*	
				(0.56)	
Processed × COVID-19				−0.70	
				(0.53)	
Year 2020 × Weeks 10–15					−1.44***
					(0.20)
Year 2020 × Weeks 16–21					−1.49***
					(0.23)
Year 2020 × Weeks 22–26					−1.77***
					(0.25)
Farmers Market Fixed Effects	YES	YES	YES	YES	YES
Vendor Fixed Effects	YES	YES	YES	YES	YES
Adjusted *R* ^2^	0.42	0.44	0.44	0.44	0.44
Observations	1,132	14,692	14,692	14,692	14,692

Notes: Parameter estimate (robust standard error). Robust standard errors are clustered at the vendor level. In each specification, Wald tests indicate that the fixed effect terms are jointly significant.

***Statistically significant at the 0.01 level; **Statistically significant at the 0.05 level; *Statistically significant at the 0.1 level.

In specification II, the market-week time trend coefficient indicates that average sales increase 5% per week during the first half of the year (*P* < 0.01). An increase in temperature of 1° F, ceteris paribus, increases average sales by 2%, while one inch of rainfall decreases sales by 21% (*P* < 0.01). The COVID-19 and year 2020 coefficients in specification II of −79% and 77%, respectively, are similar to the corresponding parameter estimates in specification I. However, the spring coefficient in specification II of 35% is considerably smaller than in specification I after we control for temperature and a weekly sales trend.

The spring coefficient is statistically insignificant in specification III when we also include a year-specific linear trend. Because we include this additional term, the interpretation of the market-week coefficient changes between specifications II and III. In specification III, the market-week coefficient indicates that average sales increased by 7% per week throughout the first half of 2019. We expect the time trend coefficient in 2019 to be greater than the time trend for both years since sales increased at a greater rate in 2019 than 2020 (Figure 1). Also, the COVID-19 coefficient is smaller in magnitude when we relax the common trends assumption and allow trends to vary by year. Still, the coefficient indicates that the COVID-19 pandemic led to a 64% reduction in average sales between the spring and winter in 2020 relative to 2019.

In specification IV, the “Animal × COVID” interaction term is positive with statistical significance (*P* < 0.1). The coefficient indicates that sales from animal product vendors during COVID-19 were 177% greater than sales from alcohol, plants, and other vendors during the non-pandemic time frame. In specification V, the effect of COVID-19 on sales was greatest during the May 31–July 4 time period, when it corresponded to an 83% decrease from the winter relative to 2019 (*P* < 0.01). These percentage decreases were 76% and 77% during the March 9–April 18 and April 19–May 30 time frames, respectively.

## 5. Discussion

The negative impact of the pandemic is consistent with negative projections of COVID-19’s impact on U.S. farmers market sales (resourcED, 2020; Thilmany et al., 2020b) and other evidence compiled by practitioners (CFSA, 2020; FMC, 2020; WSFMA, 2020). Perhaps, the biggest challenge confronting these larger neighborhood markets was the longer wait times associated with single point-of-entry systems, which likely made attending farmers markets impractical for some. The second challenge confronting vendors at these markets could have occurred from larger order sizes they needed to adhere to pre-ordering and prepackaging requirements. For instance, orchard vendors stopped selling individual apples and instead sold them by the bag, and some produce vendors started selling larger CSA style boxes instead of allowing shoppers to select vegetables on-site. So, shoppers may have been reluctant to make these purchases if the order sizes were too large or if they had quality concerns with purchasing food sight unseen.

The negative coefficient on precipitation indicates that neighborhood shoppers are less likely to patronize markets when it is raining. The positive coefficient on temperature indicates that shoppers are less likely to attend markets during the winter and spring when it is cold. Washington, D.C., had a hot summer in 2020, and we would expect a negative coefficient on temperature for summer and fall months. Similarly, the market-week time trend coefficient is positive because of the time interval we examine, and this term would presumably be negative in the second half of the year (Figure 1).

Dairy, meat, and seafood vendors experienced increases in spring sales at neighborhood markets that remained open (Tables 2 and 4). The increase in meat sales was likely attributable to supply shortages consumers confronted at traditional retail outlets as COVID-19 outbreaks closed industrial meatpacking facilities (Tonsor and Schulz, 2020). Dairy product sales at farmers markets could also have increased if these products were less available at retail outlets, although the causes of dairy stockouts would likely have been due to hoarding behavior (Lusk and McCluskey, 2020). Seafood sales may have increased as opportunities for shoppers to consume seafood in restaurants declined or if local meat vendors were unable to meet demand (Richards and Vassalos, 2020), and shoppers purchased seafood as a substitute protein instead.

Vendors that sold processed foods, plants, and other products experienced relatively large decreases in sales, with declines that ranged between 33% and 62% (Table 2). Even though snack and processed food sales increased during the pandemic (Lusk and McCluskey, 2020), they could have declined at markets if shoppers instead made such purchases in bulk at traditional retailers.

## 6. Conclusion

Our analysis of FRESHFARM’s unique database provides timely insights into local food market impacts that are not available from other sources. Overall, our findings are inconsistent with claims that local food markets are “doing relatively well” during COVID-19 (Pollan, 2020). In 2020, the pandemic debilitated weekday markets in business districts in the Washington, D.C. area, and had a particularly devastating impact on the prepared food vendors at those markets. Even for the neighborhood markets that remained open, vendors experienced a loss of sales from social distancing regulations. However, at these neighborhood markets, alcohol, dairy, meat, and seafood vendors fared relatively well (Table 2).

The limitations of our study are associated with its narrow geographic area, market channel scope, and time frame. USDA’s surveys, like the Local Food Marketing Practices Survey and Agricultural Resource Management Survey, will eventually provide the most comprehensive national-level information about COVID-19’s impact on DTC farms and ranches (although not non-farming DTC vendors). First, USDA will collect data from DTC farms for 2020 in its entirety, whereas we focus on the first half of 2020. Second, USDA’s data will document how DTC farms fared nationally. Since FRESHFARM is a relatively high-capacity farmers market organization that implemented many innovations to adapt to the pandemic, lower capacity markets may have experienced greater declines in sales than FRESHFARM. For instance, it may not have been possible for rural farmers market vendors to have quickly developed online platforms if they did not have high-speed Internet access.

Third, USDA’s data will show the impact of COVID-19 on all local food market channels. Social distancing guidelines implied that local producers experienced abrupt declines in non-DTC sales to away-from-home outlets that closed or curtailed operations (e.g., schools, colleges, restaurants, and hotels). So, DTC market channels for at-home food purchases may have performed better than non-DTC channels. Also, other DTC market channels may have performed better than farmers markets. For instance, some vendors may have implemented home delivery operations in response to challenges with selling at farmers markets (Thilmany et al., 2020a).

Fourth, our study does not measure how COVID-19 impacted the financial status of either farmers markets vendors or organizations. While sales for some vendors may have increased, their costs may have increased also for the reasons we described earlier. Also, COVID-19 has increased expenses for farmers market managers. These costs arise from buying protective equipment for market staff, adding staff to enforce social distancing guidelines, and other operational costs (FMC, 2020). A simultaneous decline in revenue from vendors and increase in costs imply that markets are confronting challenges maintaining balanced budgets. If these budget deficits become persistent, it could lead to a permanent reduction in the number of U.S. farmers markets.

## Data Availability

The primary data set used in this paper is FRESHFARM’s proprietary data, and it cannot be made publicly available.

## Footnotes

1 Food prices increased sharply during the spring of 2020. So, for all sales data, we convert dollar values of DTC sales to July 2020 dollar values using the food consumer price index (BLS, 2020).

2 In 2019, these market-weeks correspond to March 10–July 6 and January 1–March 9, respectively.

3 Of the 193 vendors that sold at these 7 markets in the first half of either 2019 or 2020, 46 participated in 2019 only and 23 participated in 2020 only. Our results are effectively unchanged when we only include observations from the 124 vendors that sold at these markets in both 2019 and 2020. This robustness check indicates it is unlikely that vendor turnover that could potentially be unrelated to COVID-19 is influencing the results.

4 This increase in winter sales is partially due to earlier openings in 2020 of the Monroe Street and Mosaic markets relative to 2019 (Table 2). Regardless, only 17% of the observations come from these two markets (Table 3), and the regression results are effectively unchanged irrespective of whether we include or exclude observations from these two markets.

5 The impacts that we report in this section represent the percentage change in sales from an increase of one unit for the corresponding independent variable. Since we are estimating log-linear regressions, for each parameter  in Table 4, this percentage change equals 100*(−1).

## Supplementary material

For supplementary material accompanying this paper visit https://doi.org/10.1017/aae.2020.37. click here to view supplementary material
