# Supplementary material for: COVID-19’s Impact on Farmers Market Sales in the Washington, D.C., Area
Source: Journal of Agricultural and Applied Economics. 2021 Jan 13:1–16. doi: 10.1017/aae.2020.37 (PMC7900660; doi:10.1017/aae.2020.37)
Supplement: Supplementary file 1 [file S1074070820000371sup001.pdf]

Appendix 1. Timeline of relevant social distancing guidelines in the Washington, D.C., area

Date	Development
March 19 to March 24	Maryland and Washington, D.C., deem farmers markets as essential businesses. They are encouraged to stay open and adopt social distancing guidelines.
March 24	Virginia does not deem farmers markets as essential businesses. To remain open, they must eliminate on-site food consumption, on-site browsing, and congregation points.
March 30	Maryland, Virginia, and Washington, D.C., issue shelter-in-place orders to residents.
April 8	Washington, D.C., reverses course, and deems farmers markets and fish markets as non-essential businesses. Markets must apply for a waiver to reopen each week, which is contingent on the Washington, D.C., government approving their proposed social distancing protocols.
May 15	Virginia allows farmers markets to reinstitute on-site shopping, while retaining social distancing protocols.
May 29 to June 1	Washington, D.C., metropolitan area implements a "phase 1" reopening of the economy (e.g., restaurants can permit outdoor dining).
May 29 to June 1	Protests about George Floyd and police brutality occur in Washington, D.C.
June 12 to June 22	Washington, D.C., metropolitan area implements a "phase 2" reopening of the economy (e.g., restaurants can permit indoor dining at 50% capacity).
